# Investigating the impact of antibiotic-induced dysbiosis on protection from *Clostridium difficile* colitis by mouse colonic innate lymphoid cells

**DOI:** 10.1128/mbio.03338-23

**Published:** 2024-02-20

**Authors:** Md Jashim Uddin, Brandon Thompson, Jhansi L. Leslie, Casey Fishman, Katia Sol-church, Pankaj Kumar, William A. Petri

**Affiliations:** 1 Division of Infectious Diseases and International Health, Department of Medicine, University of Virginia School of Medicine, Charlottesville, Virginia, USA; 2 Arcus Biosciences, Hayward, California, USA; 3 Genome Analysis and Technology Core, University of Virginia School of Medicine, Charlottesville, Virginia, USA; 4 Department of Biochemistry and Molecular Genetics, University of Virginia School of Medicine, Charlottesville, Virginia, USA; University of Arizona, Tucson, Arizona, USA

**Keywords:** *Clostridioides difficile*, innate lymphoid cells, colitis, single-cell RNAseq, host immune response

## Abstract

Innate lymphoid cells (ILCs) play a critical role in maintaining intestinal health in homeostatic and diseased conditions. During *Clostridium difficile* infection (CDI), IL-33 activates ILC2 to protect from colonic damage and mortality. The function of IL-33 and ILC is tightly regulated by the intestinal microbiota. We set out to determine the impact of antibiotic-induced disruption of the microbiome on ILC function. Our goal was to understand antibiotic-induced changes in ILC function on susceptibility to *C. difficile* colitis in a mouse model. We utilized high-throughput single-cell RNAseq to investigate the phenotypic features of colonic ILC at baseline, after antibiotic administration with or without IL-33 treatment. We identified a heterogeneous landscape of colonic ILCs with gene signatures of inflammatory, anti-inflammatory, migratory, progenitor, plastic, and antigen-presenting ILCs. Antibiotic treatment decreased ILC2 while coordinately increasing ILC1 and ILC3 phenotypes. Notably, Ifng^+^, Ccl5^+^, and Il23r^+^ ILC increased after antibiotics. IL-33 treatment counteracted the antibiotic effect by downregulating ILC1 and ILC3 and activating ILC2. In addition, IL-33 treatment markedly induced the expression of type 2 genes, including Areg and Il5. Finally, we identified amphiregulin, produced by ILC2, as protective during *C. difficile* infection. Together, our data expand our understanding of how antibiotics induce susceptibility to *C. difficile* colitis through their impact on ILC subsets and function.

**IMPORTANCE:**

*Clostridium difficile* infection (CDI) accounts for around 500,000 symptomatic cases and over 20,000 deaths annually in the United States alone. A major risk factor of CDI is antibiotic-induced dysbiosis of the gut. Microbiota-regulated IL-33 and innate lymphoid cells (ILCs) are important in determining the outcomes of *C. difficile* infection. Understanding how antibiotic and IL-33 treatment alter the phenotype of colon ILCs is important to identify potential therapeutics. Here, we performed single-cell RNAseq of mouse colon ILCs collected at baseline, after antibiotic treatment, and after IL-33 treatment. We identified heterogeneous subpopulations of all three ILC subtypes in the mouse colon. Our analysis revealed several potential pathways of antibiotic-mediated increased susceptibility to intestinal infection. Our discovery that Areg is abundantly expressed by ILCs, and the protection of mice from CDI by amphiregulin treatment, suggests that the amphiregulin-epidermal growth factor receptor pathway is a potential therapeutic target for treating intestinal colitis.

## INTRODUCTION

Innate lymphoid cells (ILCs) are the innate counterpart of T-helper cells. Similar to Th cells, ILCs are regulated by the transcription factors Tbet (ILC1), GATA-3 (ILC2), and RORγT (ILC3) and secrete type 1, type 2, and type 3 cytokines, respectively, during homeostatic and inflammatory conditions (1–4). ILCs are found as tissue-resident cells in various tissues, including the lung, liver, mesenteric lymph nodes, skin, bone marrow, spleen, and gut, where they function to maintain tissue integrity (5–8). ILCs play a critical role in maintaining intestinal homeostasis, and the balance of ILCs is altered in the colon during experimental or infection-induced colitis (9–11). ILC3 production of IL-23 is associated with intestinal pathology (12–15). The importance of ILCs in protection from *Clostridium difficile* infection (CDI) has been shown in a mouse model, where Rag^−/−^ γC^−/−^ mice (lacking ILCs in addition to T and B cells) had increased susceptibility compared to Rag^−/−^ mice (deficient in T and B cells but with an intact ILC compartment) (16, 17).

Intestinal commensal bacteria maintain continuous interaction with mucosal immune cells and shape their function. The intestinal bacteria directly or indirectly regulate ILC function, potentially via toll-like receptors on ILCs (18, 19) or by microbiota effects on intestinal epithelial cells, dendritic cells, or macrophages (20). For example, intestinal bacteria release short-chain fatty acids and secondary bile acids that can activate ILC responses during intestinal inflammation (21, 22).

Antibiotic disruption of the intestinal microbiota impacts the otherwise healthy microbiota-immune interaction. Antibiotic treatment is a major risk factor for several colonic diseases, including *C. difficile* and amebic colitis (17, 23, 24). One of the mechanisms by which antibiotics increase susceptibility to colitis is by decreasing the level of IL-33 in the colon. The regulation of intestinal IL-33 by microbiota has been further established by the fact that fecal-microbiota transplantation rescues antibiotic-associated depletion of IL-33 (17). Our group discovered that IL-33 protects mice from intestinal epithelial disruption and mortality caused by CDI without altering bacterial burden in the colon (17). This protection was mediated by the activation of ILC2s, and the adoptive-transfer of ILCs was sufficient to protect ILC-deficient mice from *C. difficile*-associated mortality (16, 17, 25). There are limited data available on how antibiotics and IL-33 treatment impact the ILC compartment in the colon.

How ILC2s protect mice from CDI has yet to be fully understood. At homeostasis or after stimulation, ILC2 could mediate protection via the production of type 2 cytokines, including IL-13, IL-5, and IL-4, and in addition via stimulation of the epidermal growth factor receptor (EGFR) by amphiregulin (16, 19, 26). Amphiregulin has been reported to function downstream of the IL-33 pathway to repair epithelial damage caused by dextran sodium sulfate (DSS)-induced colitis (27). The role of amphiregulin in CDI; however, has not been investigated.

In this study, we performed single-cell RNAseq analysis on mouse colonic ILCs collected at baseline, after antibiotic treatment, and after IL-33 remediation of the impact of antibiotics on *C. difficile* susceptibility. We observed heterogeneity in all three subtypes of ILCs, with ILC2s as the predominant population. Treating with antibiotics increased ILC1 and ILC3 clusters while decreasing ILC2 clusters. There were also functional changes in ILCs upon antibiotic treatment, with increased expression of Ifng and Ccl5 in ILC1, and Il23r in ILC3s. Our data suggest that antibiotics might increase susceptibility to CDI in part by inducing Il23r^+^ ILC3s, as IL-23 signaling has been found to potentiate disease severity in CDI (28). Additionally, we discovered that IL-33 treatment downregulated ILC1 and ILC3 responses. Interestingly, IL-33 treatment did not impact all the ILC2 clusters similarly. In some clusters of ILC2s, there was a dramatic increase in cell numbers compared to the rest of the ILC2 clusters. We observed a robust expression of Il5 and Areg at baseline and during IL-33 treatment, with antibody neutralization of amphiregulin increasing susceptibility. In summary, the phenotyping of ILC populations provided insight into potential mechanisms of antibiotic-induced susceptibility to CDI via alteration of ILC function.

## RESULTS

### ILC subsets are heterogeneous in the mouse colon as determined by single-cell RNAseq

**Fig 1:**
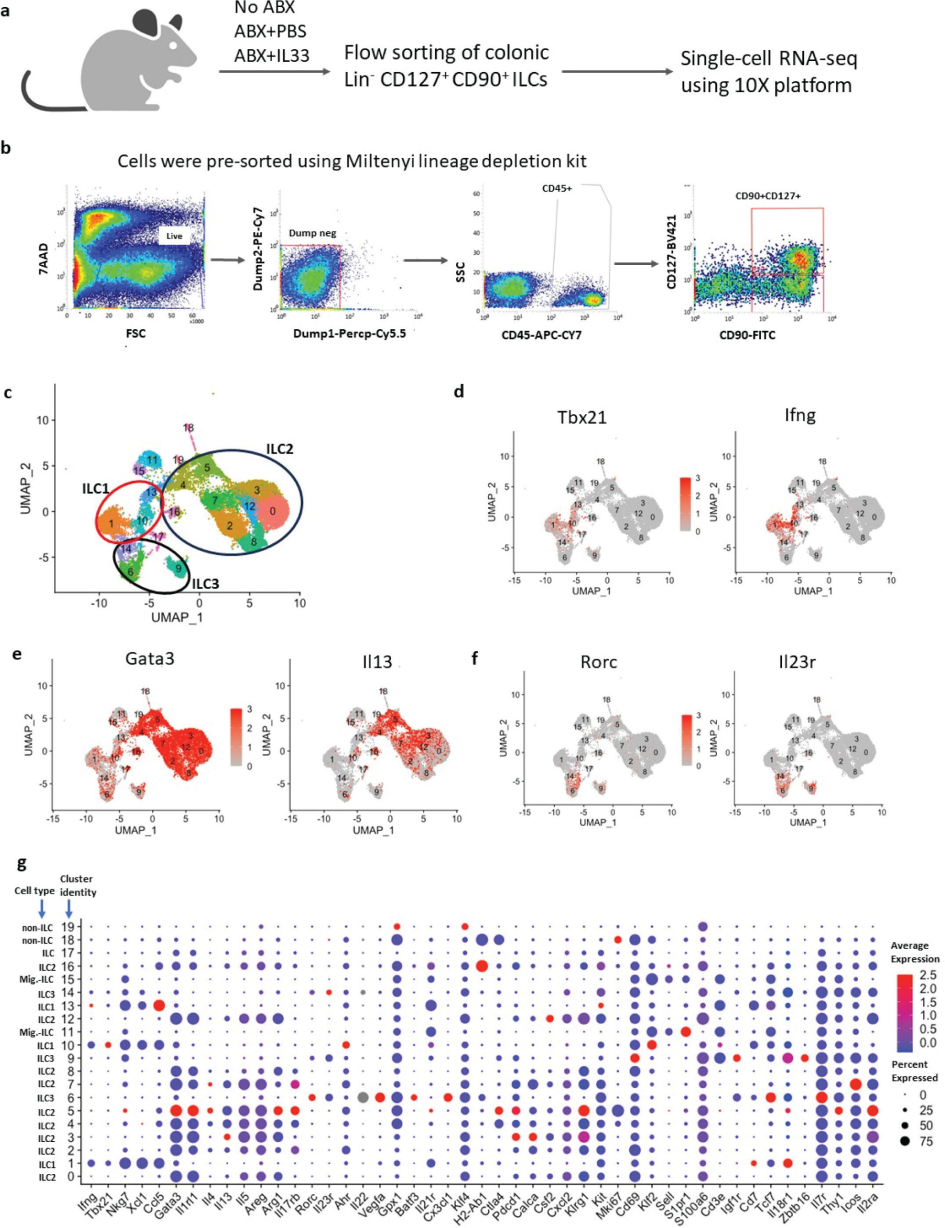
ILC subsets are heterogeneous in the mouse colon. Miltenyi lineage depletion kits were used to initially purify lineage-negative cells from the colon of 8-weeks-old C57Bl/6J mice. Lin^−^CD45^+^CD127^+^CD90^+^ ILCs were then sorted, followed by single-cell RNAseq analysis using the 10× genomics platform. (a) Experimental design. (b) Gating strategy for flow sorting of ILCs. (c) Cell cluster visualization using uniform manifold approximation and projection (UMAP) after dimensional reduction. (d, e, and f) Expression profile of master transcription factors and a marker protein to identify ILC1, ILC2, and ILC3 clusters. (g) Dot plot to visualize the expression of ILC-associated genes. ABX, antibiotics; Mig-ILC, migratory ILC; c, d, e, f, and g, *N* = 16,683 cells pooled from 30 mice.

To investigate how antibiotics and IL-33 treatment can shape colonic ILC function, we performed single-cell RNAseq analysis on 16,683 colonic ILCs collected at steady state and after antibiotic treatment and IL-33 treatment (Fig. 1a). To increase the purity of colonic ILCs we performed a two-step purification method. At first, we used the Miltenyi direct lineage depletion kit to efficiently deplete T cells, B cells, monocytes/macrophages, granulocytes, erythrocytes, and their precursors from the cell mixtures. We then flow-sorted lineage-negative CD45^+^CD127^+^CD90^+^ ILCs (Fig. 1b). We generated single-cell RNAseq profiles of these 16,683 cells utilizing a droplet-based 10× genomics platform. The quality of data was tested by examining unique molecular identifiers (UMI) per cell, genes detected per cell, UMIs vs genes detected, and the percent of mitochondrial genes (Fig. S1). Dimensionality reduction was performed using “Seurat” packages in “R.”

The uniform manifold approximation and projection (UMAP) clustering revealed colonic ILCs were heterogeneous, with 20 distinct clusters (Fig. 1c). Clusters with less than 50 cells were excluded from the analysis. We analyzed the expression profile of key transcription factors for ILC1, ILC2, and ILC3 (Tbx21, Gata3, and Rorc, respectively) and other marker genes including Ifng, Il13, and Il23r to identify ILC subsets in these clusters (29) (Fig. 1d, e, and f). Based on the expression profiles of these key genes which were previously used to determine ILC subsets (29), we classified clusters 1, 10, and 13 as ILC1, clusters 0, 2, 3, 4, 5, 7, 8, 12, and 16 as ILC2, and clusters 6, 9, and 14 as ILC3 (Fig. 1c). We additionally analyzed the expression profile of 47 genes involved in ILC activation, maturation, proliferation, differentiation, and/or migration (Fig. 1g) (30, 31). The dot plot shows the heterogeneous expression of these 47 genes in colonic ILCs (Fig. 1g).

Among the three ILC1 clusters, cluster 13 had fewer cells that expressed Tbx21 or Ifng although all three clusters had robust expression of effector genes including Nkg7, Xcl1, and Ccl5. These three ILC1 clusters also had variable expression of other genes including Il21r, Cd7, and Il18r1. Interestingly, cluster 13 was positive for Cd3e and Cd7 previously found to be expressed by bone marrow ILCs (31). This suggested that cluster 13 is dominated by immature progenitor cells.

Among the ILC2 clusters, cluster 5 exhibited robust expression of Gata3, Il1rl1, Arg1, Rgs2, Klrg1, Id2, Il2ra, and Il17rb (Fig. 1g). Interestingly, the majority of the cluster 5 cells were Mki67^+^ indicating that these cells were proliferative. In addition, the robust expression of the IL-33 receptor (Il1rl1) and IL-25 receptor (Il17rb) in cluster 5 suggests it would preferentially respond to IL-33 and IL-25. Except for cluster 16, the majority of ILC2 clusters showed robust expression of Areg and IL-5. However, Arg1, Il4, and Il13 were variably expressed by ILC2 clusters.

Among three ILC3 clusters, cluster 6 had the highest expression of Vegfa, Rorc, and Il23r, but the lowest expression of csf2. Interestingly, cluster 6 also expressed Igf1r, which was previously shown to be associated with cell plasticity (31). Cluster 6 was the only cluster that was positive for Cx3cl1, an important chemokine for the recruitment of Cx3cr1^+^ inflammatory immune cells. Cluster 9 was the only cluster that expressed Zbtb16. Zbtb16 was found to be abundant in bone marrow ILCs (31). The presence of Batf3^+^ cells emphasized the differentiation capability of ILC3s.

Il7r (CD127) was used as one of the surface markers to sort total ILCs, which is a canonical lymphoid cell marker (32). Clusters 18 and 19 were the smallest clusters and fewer cells in these clusters were Il7r^+^ (Fig. 1g; Fig. S2). This suggests that these clusters could be due to non-lymphoid contamination of the cells. The clusters 11, 15, and 17, although Il7r^+^, did not express any ILC marker genes (Fig. 1d, e, and f; Fig. S2). Among them, clusters 11 and 15 displayed predominant expression of S1pr1 (sphingosine-1-phosphate receptor 1) and Sell (Selectin L), which are associated with cell trafficking. Clusters 11 and 15 were also positive for Tcf7 which is a marker for naïve ILCs, suggesting that they have the capability to migrate to other tissues and to differentiate (Fig. 1g) (31, 33). All of the ILC1 and ILC3 clusters had Tcf7^+^ and Il18r1^+^ cells which are known signatures for ILC progenitors (Fig. 1g). This suggests that colonic ILC1s and ILC3s can be transdifferentiated upon external stimulation or during infection. Recently, antigen-presenting ILCs were demonstrated in the central nervous system and small intestine (34, 35). Cluster 16 exhibited abundant expression of H2-Ab1 revealing the involvement of colonic ILCs in regulating T cell function with their antigen-presentation ability (Fig. 1g). Altogether, our data revealed that colonic ILCs were a heterogeneous population with diverse functional signatures.

### Antibiotics induced ILC1 and ILC3 responses and decreased ILC2

**Fig 2:**
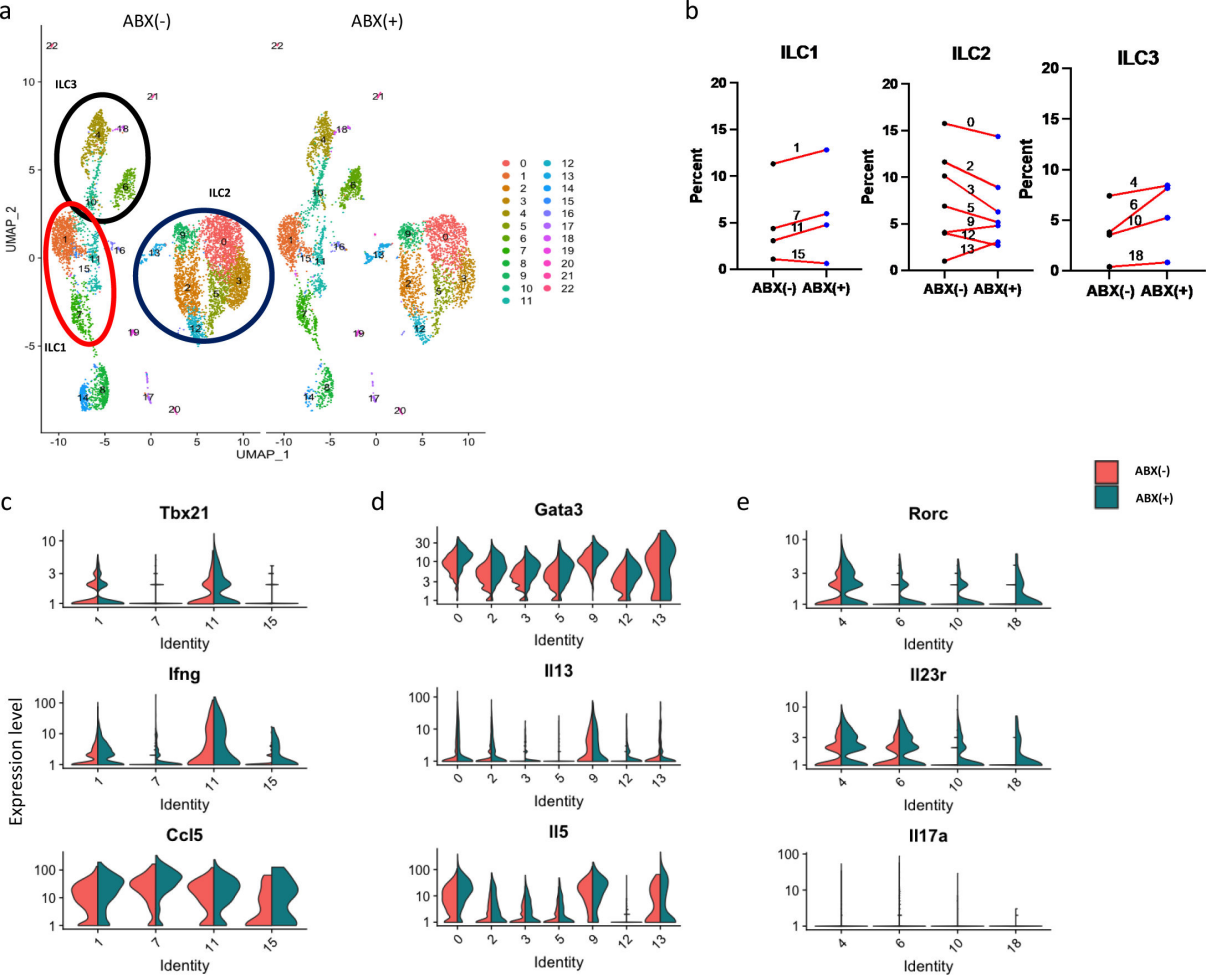
Antibiotics reduced ILC2s and upregulated ILC1 and ILC3 clusters. Eight-weeks-old C57BL/6J mice were treated with an antibiotic cocktail of metronidazole, vancomycin, gentamycin, and colistin administered in drinking water and clindamycin via i.p. injection. scRNAseq analysis was conducted as described. (a) Cell cluster visualization using UMAP at ABX (−) and ABX (+) treatment conditions. (b) The ratio of ILC1, ILC2, and ILC3 clusters. (c, d, and f) Expression profile of (c) type 1 genes (Tbx21, Ifng, and Ccl5), (d) type 2 genes (Gata3, Il13, and Il5), and (e) type 3 genes (Rorc, Il17a, and Il23r).

Exposure to antibiotics is the primary risk factor for both hospital-acquired and non-hospital-acquired CDI (36, 37). The mouse model of CDI relies on treatment of mice with antibiotics prior to *C. difficile* infection (17, 24, 38). To understand the impact of antibiotics on colonic ILCs, we tested for changes in ILCs upon treatment with antibiotics. The re-clustering of ILCs from antibiotic-treated and non-treated mice identified 4 ILC1, 7 ILC2, and 4 ILC3 clusters (Fig. 2a; Fig. S4).

Antibiotics decreased the ratio of five different ILC2 clusters (clusters 0, 2, 3, 5, and 12) (Fig. 2b, middle figure) while cluster 9 and cluster 13 increased. In the UMAP (Fig. 2a), cluster 13 is located separately from other ILC2 clusters and closer to ILC1 and ILC3 clusters. This suggests that ILC2s in cluster 13 might function differently than other ILC2s. This functional difference was also suggested by the fact that, unlike other clusters of ILC2s, cluster 13 expressed Ccl5 and Ccr5, which are mostly observed in ILC1s (Fig. S3). Although we observed a reduction in ILC2 numbers with antibiotics, the expression of type 2 genes was not reduced (Fig. 2d; Fig. S5b). Rather, we observed increased expression of Areg, Arg1, and Il13 in ILC2 cluster 0 (Fig. S5b).

In contrast to ILC2s, antibiotics increased most of the ILC1 and ILC3 clusters (Fig. 2b). Antibiotics increased the number of Ifng^+^ cells in clusters 7 and 15 (Fig. 2c; Fig. S5a) and upregulated the expression of Nkg7 in cluster 1 and expression of Xcl1, Ccl5, Ifng, and Il21r in cluster 7 (Fig. S5a). After antibiotics, the number of Rorc^+^ and Il23r^+^ ILC3s was markedly increased (Fig. 2e). Especially, clusters 6, 10, and 18 had few or no Rorc^+^ and Il23r^+^ cells at homeostatic condition but noticeably higher numbers of Rorc^+^ and Il23r^+^ cells during antibiotic treatment (Fig. 2e; Fig. S5c). Altogether, our data showed antibiotics upregulated ILC1 and ILC3 phenotypes while decreasing the number of ILC2s.

### IL-33 treatment downregulated the antibiotic-induced ILC1 and ILC3 responses and upregulated ILC2s

**Fig 3:**
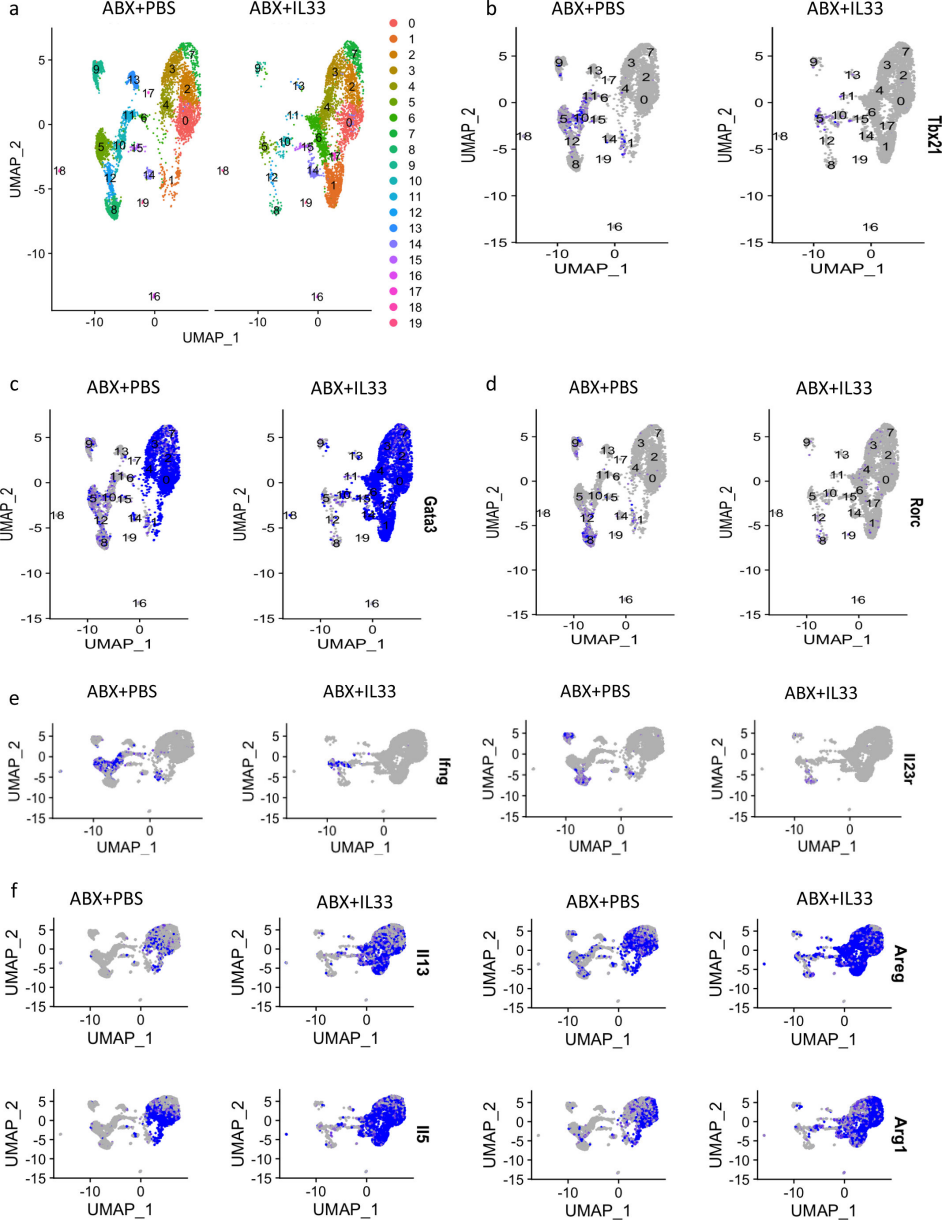
IL33 treatment downregulated ILC1s and ILC3s and upregulated ILC2s. (a) UMAP clustering of colonic ILCs from ABX+PBS and ABX+IL-33 treated mice. (b) Expression of Tbx21, (c) Gata3, (d) Rorc. (e and f) Comparing the expression of Ifng, Il23r, Il5, Il13, Areg, and Arg1 between ABX+PBS and ABX+IL-33 treated mice. ABX, antibiotics.

The role of the IL-1 family cytokine IL-33 during CDI has been investigated by our group (1). IL-33 acted via ILC2s to protect mice from CDI mortality and weight loss. However, the downstream mechanisms of ILC2-mediated protection from CDI were not understood, nor the impact of IL-33 on other ILCs. Here, we compared the transcriptional profile of colonic ILCs from antibiotic-treated mice that received IL-33 or phosphate-buffered saline (PBS) treatment. Re-clustering of ILCs from IL33 vs PBS treatment conditions generated 19 different clusters (Fig. 3a).

Based on the expression profile of Tbx21, Gata3, and Rorc (Fig. 3b, c, and d) clusters 0, 1, 2, 3, 4, 6, 7, 14, and 15 were ILC2s and clusters 5, 8, 9, 10, 11, and 12 were ILC1s and ILC3s. With IL-33 treatment, there was a reduction in all ILC1 and ILC3 clusters (Fig. 3a, b, and d). IL-33 not only impacted the size of ILC1 and ILC3 clusters but also reduced the expression of Ifng and IL23r (Fig. 3e). Another IL-1 family cytokine IL-18 has been implicated in inducing colitis by limiting goblet cell differentiation (39). While Il18 was not expressed by any ILCs, ILC1, and ILC3 expressed Il18r1, and with the IL-33 treatment, the expression of Il18r1 decreased (Fig. S6). These data suggest IL-33 treatment in mice downregulated ILC1 and ILC3 responses in the colon.

IL-33 treatment did not modulate all ILC2 clusters equally. We observed dramatic enrichment of cells in clusters 1, 4, and 6 with IL-33 (Fig. 3a). In addition, we investigated the expression of major type 2 proteins in colonic ILCs. During IL-33 treatment, we observed dramatic induction of the expression of Il5, Il13, Areg, and Arg1 (Fig. 3f). Altogether, our data suggest that IL-33 treatment dampened ILC1 and ILC3 phenotypic signatures while inducing ILC2s.

### Amphiregulin signaling protects from *C. difficile*-associated disease

**Fig 4:**
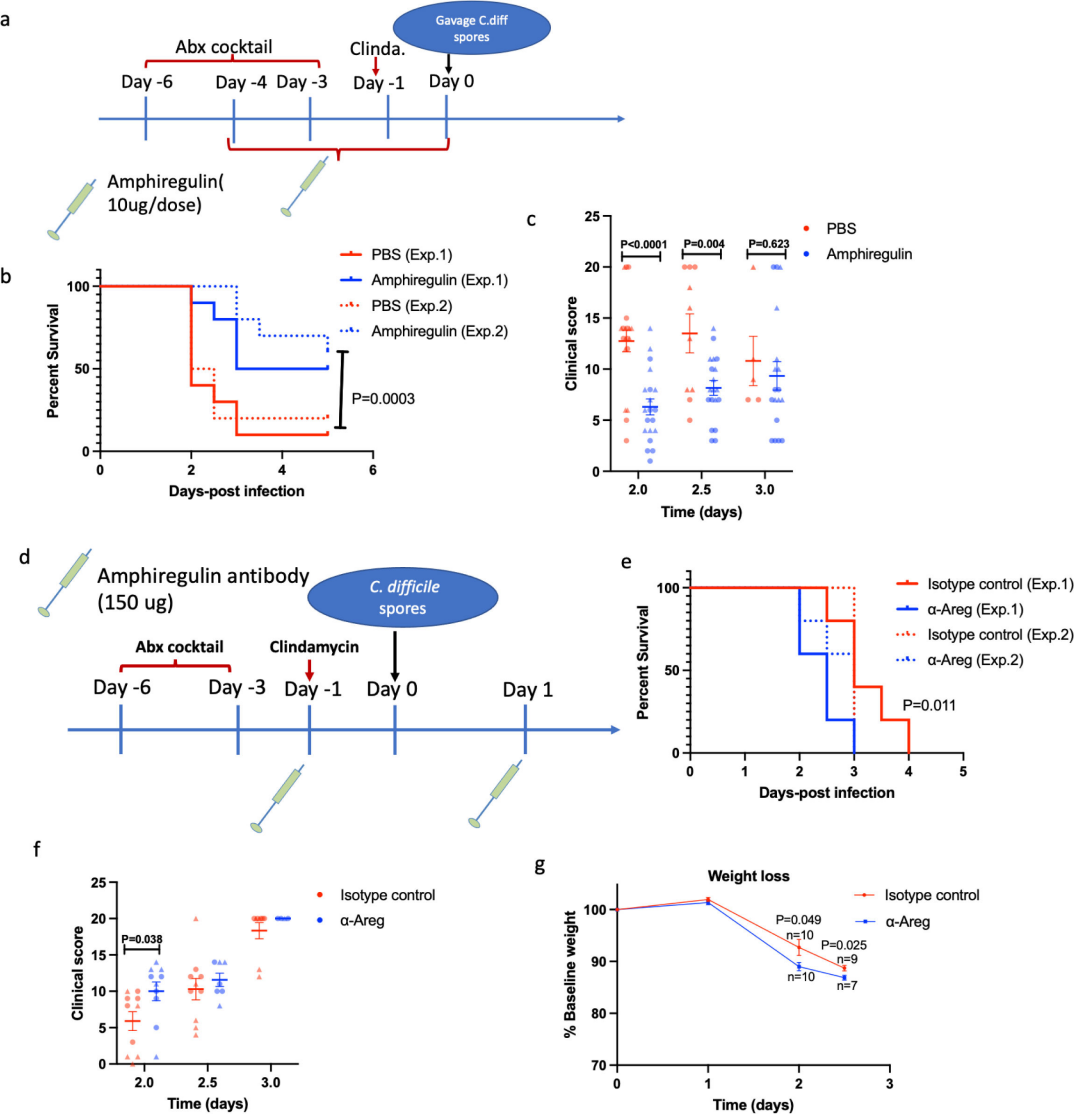
Amphiregulin protects from mortality during *C. difficile* infection. Eight- to ten-weeks-old C57BL6/J mice were treated with antibiotics and then infected with 1,000 *C. difficile* spores via oral gavage. (a) Experimental design and amphiregulin treatment. (b) Survival and (c) clinical scores after infection. (d) Experimental design for amphiregulin antibody treatment. (e) Survival, (f) clinical scores, and (g) weight loss after anti-amphiregulin treatment. (b and e) Statistical significance was determined by log-rank test (b, *n* = 20 mice in each group; e, *n* = 10 mice in each group). (c and f) Circles and triangles represent mice from two separate experiments. (c, f, and g) Statistical significance was determined by *t*-test. Error bars indicate SEM.

Our single-cell RNAseq analysis of colonic ILCs revealed the abundant expression of Il5 and Areg by ILC2s (Fig. 1g; Fig. 3f). Besides, with the IL-33 treatment there was a dramatic induction of Il5 and Areg expression. Our group discovered a protective role of IL-5 during *C. difficile* infection (40). The role of amphiregulin during *C. difficile* infection had not been tested. Amphiregulin is known to contribute to intestinal epithelial regeneration (26), and IL-33 was shown to activate the amphiregulin-EGFR pathway to repair intestinal damage during DSS colitis (27). We asked whether increasing amphiregulin by treating mice with recombinant amphiregulin had an impact on CDI. To test the role of amphiregulin, 10 µg of recombinant amphiregulin was administered intraperitoneally for 5 days (Fig. 4a). Amphiregulin-treated mice had significantly reduced mortality (Fig. 4b) and reduced clinical scores (Fig. 4c). Although the difference was not robust, inhibiting amphiregulin conversely increased susceptibility, as demonstrated by earlier mortality in mice treated with 150 µg of anti-amphiregulin antibody on day −1 and day +1 of *C. difficile* challenge (Fig. 4d and e). For both the recombinant amphiregulin and the antibody treatment, we followed the timing used by our group previously (17, 28). While the difference was modest, the anti-amphiregulin-treated mice had higher clinical scores on day 2 and weight loss on days 2 and 2.5 (Fig. 4f and g). We concluded that IL-33 induction of Areg expression could be a downstream mechanism of ILC2 protection.

## DISCUSSION

Here, we generated a transcriptomic profile of flow-sorted mouse colon ILCs using single-cell RNAseq. The study revealed the heterogeneous signatures of murine colonic ILCs and the alteration of their functional signatures after antibiotic and IL-33 treatment. Broad-spectrum antibiotics potentiated ILC1 and ILC3 signature genes while decreasing ILC2s. We also observed that supplementing antibiotics-treated mice with recombinant IL-33, a cytokine suppressed by antibiotics, decreased ILC1 and ILC3 and increased ILC2. Finally, we noted the abundant expression of Areg by all ILCs at baseline and during IL-33 treatment, and showed that amphiregulin signaling was protective for CDI.

Heterogeneous gene signatures among colon ILCs signify functional diversity. ILCs that maintain tissue integrity during homeostatic conditions might be different from the ILCs that respond to external stimulation or combat pathogenic encounters. We observed that ILCs displaying migratory gene signatures lacked the expression of ILC subtype-specific marker genes. This implies that migratory ILCs may have the ability to traffic and play a crucial role in inflammatory settings rather than functioning during homeostatic conditions. Depending on the external stimulation or diseased tissues, ILCs can proliferate to increase their numbers or can transdifferentiate to become another type of ILC (41). These trans-differentiation and proliferative natures are common among colon ILCs. We observed a higher percentage of cells in ILC1 and ILC3 than in ILC2 clusters showed progenitor signatures (Cd3e, Igf1r, Cd7, Tcf7, and Il18r1) (Fig. 1g). ILC1s and ILC3s might be more efficient in trans-differentiation. This is further justified by the fact that during IL-33 treatment, all the ILC1 and ILC3 but few of the ILC2 clusters were altered (Fig. 3a).

Antibiotics increase susceptibility to CDI by disrupting cross-talk between the microbiome and mucosal immune system, reducing IL-33 and IL-25 expression and creating a pro-inflammatory environment that favors CDI (25). Consistent with this, we observed increased expression of Ifng and Ccl5 in ILC1s and Il23r in ILC3s during antibiotic treatment. Alteration in ILCs could be a potential mechanism of antibiotic-induced susceptibility, as supported by the evidence that colitis can be reversed by inhibiting IL-23 signaling (28). A previous study showed that Ifng^+^ ILC1s were protective during CDI (16). That study ablated Ifng^+^ or Tbet^+^ ILC1s in mice and found that those mice were more susceptible to CDI. While this study showed that a baseline level of ILC1 is necessary, it is possible that upregulation of ILC1 accompanied by increased ILC3 due to antibiotics could create a pro-inflammatory environment and accelerate tissue damage. An endogenous level of IFNγ might protect from *C. difficile* by clearing bacterial burden; however, excessive production of IFNγ could cause collateral damage to host tissue. Besides, antibiotic-activated ILC1s could be phenotypically different from endogenous ILC1s. Because IL-33 mediated protection from *C. difficile* is accompanied by a reduction of ILC1s and ILC3s and activation of ILC2s (17), it would be important to test if ILC-specific inhibition of Ifng, Ccl5, and Il23r would protect antibiotic-treated mice from CDI.

Although ILC3 is known to contribute to maintaining tissue integrity via IL-22, IL-17a, and Granulocyte macrophage colony-stimulating factor (GM-CSF), several studies found that IL23-responsive ILC3 was associated with intestinal pathology (7–9, 33, 34). In accordance with these reports, our lab found that IL-23 signaling contributed to disease severity and mortality during CDI (28). Our observation that antibiotics increased Il23r^+^ ILCs supports the idea that antibiotics-mediated induced susceptibility to CDI is partly due to Il23r^+^ ILC3s.

Recent studies have reported on the migratory nature of ILCs, including that small intestinal-ILC3s had the ability to migrate to mesenteric lymph nodes in a CCR7-dependent manner (42) and that the expression of migration-associated genes, including S1pr1 and Sell (43) were involved in the trafficking of small intestinal ILC2s to the lungs to promote defense against helminths (44). We observed the expression of S1pr1 and Sell in some clusters of colonic ILCs (Fig. 1g, clusters 11, 15, and 16). However, these S1pr1 and Sell expressing ILCs did not express markers of any of the three ILC subtypes but co-expressed Tcf7, which is a marker of ILC progenitors (Fig. 1g). This indicates that these colonic ILC clusters may have the ability to traffic other tissues and become any ILC subtypes.

It is possible that some ILC2 clusters are more important during IL-33-mediated protection from CDI compared to other ILC2 clusters. We observed three ILC2 clusters (Fig. 3a, clusters 1, 4, and 6) increase dramatically during IL-33 treatment compared to the others. This enrichment might be contributed by cells from ILC1 and ILC3 clusters. The reduction of ILC1 and ILC3 clusters and the enrichment of ILC2 clusters during IL-33 treatment suggests that ILC populations are plastic, and depending on the external stimulation, they can switch their phenotypes. Our data also suggest that IL-33 might protect from CDI by combined downregulation of type 3 signaling, as observed by the decreased expression of Il23r, and upregulation of type 2 signaling, as observed by the increased expression of Il5, Il13, Areg, and Arg1.

Amphiregulin is known to be a component of type 2 immunity. Studies have divulged the importance of amphiregulin in intestinal tissue regeneration and repair after chemical or infection-induced damage. Mice with a genetic deficiency of amphiregulin or amphiregulin receptor (EGFR) were found to be susceptible to DSS-induced colitis (27). Intestinal epithelial regeneration was compromised in Areg^−/−^ mice after total body irradiation (26). During IL-33 treatment, we observed that all ILC subtypes expressed Areg. By treating mice with recombinant amphiregulin and inhibiting amphiregulin with a monoclonal antibody, we showed that amphiregulin signaling reduced disease from CDI. Although ILC2s are thought to be the major source of amphiregulin, macrophages and neutrophils also release amphiregulin to aid tissue repair (45, 46). In the future, we plan to utilize ILC-specific amphiregulin knock-out mice to test if Areg^−/−^ ILC2s lose the capacity to protect Rag2^−/−^ γC^−/−^ mice. We also want to investigate if any intestinal epithelial cell types are altered in WT or Areg^−/−^ mice after *C. difficile* infection to understand the potential downstream mechanisms.

One of the limitations of our current study is that we started amphiregulin and anti-amphiregulin treatment before the *C. difficile* challenge. However, in future experiments, we plan to treat mice with amphiregulin at different time points after the *C. difficile* challenge to test how long after the infection the intervention can be effective. We are also interested in testing if treating mice with amphiregulin or anti-amphiregulin during primary CDI would impact the outcomes of the recurrent CDI.

Collectively, the transcriptomic profile we created using single-cell RNAseq is a step toward delineating the role of colonic ILCs in antibiotic-induced susceptibility to CDI. Our analysis revealed several potential pathways of antibiotic-mediated increased susceptibility to intestinal infection. Future exploration of the IL-33-ILC2 pathway has the potential to identify potential immune-mediated therapies to prevent or treat CDI and other infections for which antibiotic-induced dysbiosis increases susceptibility. The robust expression of Areg by all ILCs after IL-33 treatment and the protection of mice from CDI by amphiregulin treatment suggest that the amphiregulin-EGFR pathway is a potential therapeutic target for treating intestinal colitis.

## MATERIALS AND METHODS

### Mice

All the experiments were done using 8–10 weeks old, age, and sex-matched C57BL/6J mice. Mice were purchased from Jackson Laboratory and housed in the vivarium of the University of Virginia in specific pathogen-free conditions. Mice were provided autoclaved food and water *ad libitum*. All the procedures used for this study were approved by the University of Virginia Institutional Animal Care and Use Committee.

### Antibiotics and *C. difficile* infection

Six days before the infection, mice were administered an antibiotic cocktail (45 mg/L Vancomycin, 35 mg/L Colistin, 35 mg/L Gentamycin, and 215 mg/L Metronidazole) in drinking water for 3 days. After that, antibiotic water was replaced with regular drinking water. One day before the infection, mice were given an IP injection of Clindamycin (0.016 mg/g of body weight). Mice were infected with 1,000 spores of *C. difficile* (R20291 strain) via oral gavage. After the infection, mice were monitored daily to record clinical scores and weight loss. Details about the clinical scoring system were discussed previously (17). In brief, mice were scored based on weight loss, coat, posture, activity, eye condition, and diarrhea. For weight loss and activity, scores were from 0 to 4, and for other conditions, scores were from 0 to 3. Mice were euthanized if they lost 25% or more of their baseline weight and had an activity score of 4. A combined score of ≥14 was considered a severe illness, and mice were immediately euthanized. A dead mouse was given the highest score of 20.

### Single-cell suspension and flow sorting

The whole colon from mice was dissected and opened longitudinally, followed by washing with PBS. Tissues were then left in buffer 1 (HBSS with 25 mM HEPES and 5% FBS) until harvesting all the mice. Colon tissues were incubated at 37°C temperature with gentle shaking for 40 min to remove the epithelial layer into 40 mL buffer 2 (HBSS with 15 mM HEPES, 5 mM EDTA, 10% FBS, and 1 mM DTT). Tissue sections were cut into small pieces with a fine scissor and incubated into 5 mL digestion buffer (RPMI 1640 containing 0.17 mg/mL liberase, 30 mg/mL DNase) for 30–35 min at 37°C temperature with gentle shaking. Solutions were then passed through 100 micron and 40 micron cell strainers to collect single-cell suspensions. Suspensions were centrifuged at 500 *g* for 5 min to pellet cells, followed by resuspension into 300 mL FACS buffer (2% FBS in PBS). From the cell suspensions, ILCs were enriched using a mouse lineage cell depletion kit (Milteny Biotec) following the manufacturer’s protocol. Enriched ILC solution were stained by the following monoclonal antibodies: CD19 (cat-123133), CD5 (cat-100623), CD3 (cat-100327), FceRIa (cat-134317), CD11c (cat-117327), CD90 (cat-105305), CD11b (cat-101215), Ly6C (cat-128005), CD45 (cat-103115), Ly6G (cat-127617), and CD127 (cat-566377). Lin^−^CD45^+^CD90^+^CD127^+^ ILCs were flow-sorted to perform single-cell RNAseq.

### Single-cell RNAseq

For the single-cell RNAseq, ILCs were flow sorted at the University of Virginia flow core in PBS + 0.04% BSA solution. Cells were then centrifuged and resuspended in 60 mL PBS + 0.04% BSA solution, followed by counting using the CellDrop cell counter and checking the viability using the AO/PI viability assay (DeNovix Inc). The Genome Analysis and Technology core (RRID: SCR_018883) at the University of Virginia generated single-cell indexed libraries and performed quality control analysis. In brief, cells were passed through Chromium Next GEM chip G with barcoded gel beads to generate Gel Beads-in-emulsion (GEM). After that, barcoded cDNA was generated in GEMs. GEMs were then broken, and cDNA was cleaned up and amplified for the library preparation. Sequencing was performed using the P3-100 cycle kit. The generated BCL files were then converted to FATAq format for further analysis.

### Single-cell RNAseq analysis

Cell Ranger pipeline “count” (10× genomics) was used to map the sequencing reads (FastQ files) to the mouse transcriptome (mm10) to identify the 10× barcodes and generate gene expression count matrix. The sample-specific count matrix files were imported into the Seurat package in R for further analysis. Cells with >10% of genes expressed as mitochondrial genes, unusually low and high UMI counts, and <200 feature counts were filtered out. After removing the low-quality cells, the data were normalized using the “SCT transform” function. Following normalization, samples were integrated using the “IntegrateData” function in Seurat. An elbow plot was utilized to determine the total number of dimensions to use for clustering. UMAP and PCA were generated for dimensional reduction. With a high number of cells (16,683), a resolution value of 1 was chosen to identify more meaningful subpopulations without over-clustering the population. For each cluster, the top 20 marker genes were extracted, and their unique expression was visualized using a heatmap and dot plot. The chosen resolution parameter is justified by the unique gene expression profiles of 3–5 genes observed for each cluster. Various functions, including RunUmap, DimPlot, FeaturePlot, DotPlot, and VlnPlot, were further used to investigate gene expression profiles in different clusters under different treatment conditions.

### Amphiregulin, IL-33, and anti-amphiregulin treatment

Mice were administered 10 µg recombinant amphiregulin (R&D systems, catalog #989-AR-100/CF) or PBS through intraperitoneal injections daily for 5 days starting from days −4 of CDI (27). For the IL-33 experiment, mice were injected with 0.75 µg recombinant IL-33 (Biolegend, SanDiego, CA) or PBS for 5 days starting from days −4 of CDI intraperitoneally; this dose has been found to be protective during colitis in previous studies (17, 47). For anti-amphiregulin experiments, mice were treated with 150 µg anti-amphiregulin antibody (R&D systems, cat # MAB989) or isotype control (R&D systems, cat # MAB006) on day −1 and day +1 of infection intraperitoneally (48).

### Software and statistical analysis

Single-cell RNAseq data were analyzed using the “Seurat” package in “R.” Statistical significance for the survival curve, clinical scores, and weight loss was determined by the log-rank test and unpaired *t*-test. All the tests were done using GraphPad Prism software.

## Data Availability

The single-cell RNAseq raw data files and processed data files are deposited in the GEO. The accession number for the data is GSE254628. Any further data or reagent questions should be directed to Dr. Uddin (mu2qx@virginia.edu) and Dr. Petri (wap3g@virginia.edu).

## SUPPLEMENTAL MATERIAL

Supplemental Figures mbio.03338-23-s0001.pdf Figures S1-S6.

ASM does not own the copyrights to Supplemental Material that may be linked to, or accessed through, an article. The authors have granted ASM a non-exclusive, world-wide license to publish the Supplemental Material files. Please contact the corresponding author directly for reuse.

## References

[1] Klose CSN, Kiss EA, Schwierzeck V, Ebert K, Hoyler T, d’Hargues Y, Göppert N, Croxford AL, Waisman A, Tanriver Y, Diefenbach A. 2013. A T-bet gradient controls the fate and function of Ccr6−RORγt+ innate lymphoid cells. Nature 494:261–265. doi:10.1038/nature11813 23334414

[2] Mjösberg J, Bernink J, Golebski K, Karrich JJ, Peters CP, Blom B, te Velde AA, Fokkens WJ, van Drunen CM, Spits H. 2012. The transcription factor GATA3 is essential for the function of human type 2 innate lymphoid cells. Immunity 37:649–659. doi:10.1016/j.immuni.2012.08.015 23063330

[3] Spits H, Artis D, Colonna M, Diefenbach A, Di Santo JP, Eberl G, Koyasu S, Locksley RM, McKenzie ANJ, Mebius RE, Powrie F, Vivier E. 2013. Innate lymphoid cells — a proposal for uniform nomenclature. Nat Rev Immunol 13:145–149. doi:10.1038/nri3365 23348417

[4] Spits H, Di Santo JP. 2011. The expanding family of innate lymphoid cells: regulators and effectors of immunity and tissue remodeling. Nat Immunol 12:21–27. doi:10.1038/ni.1962 21113163

[5] Chou C, Li MO. 2018. Tissue-resident lymphocytes across innate and adaptive lineages. Front Immunol 9:2104. doi:10.3389/fimmu.2018.02104 30298068 PMC6160555

[6] Fan X, Rudensky AY. 2016. Hallmarks of tissue-resident lymphocytes. Cell 164:1198–1211. doi:10.1016/j.cell.2016.02.048 26967286 PMC4973889

[7] Wallrapp A, Riesenfeld SJ, Burkett PR, Kuchroo VK. 2018. Type 2 innate lymphoid cells in the induction and resolution of tissue inflammation. Immunol Rev 286:53–73. doi:10.1111/imr.12702 30294962 PMC7370855

[8] Meininger I, Carrasco A, Rao A, Soini T, Kokkinou E, Mjösberg J. 2020. Tissue-specific features of innate lymphoid cells. Trends Immunol 41:902–917. doi:10.1016/j.it.2020.08.009 32917510

[9] Kokkinou E, Soini T, Pandey RV, van Acker A, Theorell J, Czarnewski P, Kvedaraite E, Vandamme N, Lourda M, Sorini C, Weigel W, Carrasco A, Tibbitt CA, Schlums H, Lindforss U, Nordenvall C, Ljunggren M, Ideström M, Svensson M, Henter J-I, Villablanca EJ, Bryceson YT, Rolandsdotter H, Mjösberg J. 2023. The single-cell transcriptional landscape of innate and adaptive lymphocytes in pediatric-onset colitis. Cell Rep Med 4:101038. doi:10.1016/j.xcrm.2023.101038 37160121 PMC10213874

[10] Park CH, Lee A-R, Ahn SB, Eun CS, Han DS. 2020. Role of innate lymphoid cells in chronic colitis during anti-IL-17A therapy. Sci Rep 10:297. doi:10.1038/s41598-019-57233-w 31941937 PMC6962146

[11] Zeng B, Shi S, Ashworth G, Dong C, Liu J, Xing F. 2019. ILC3 function as a double-edged sword in inflammatory bowel diseases. Cell Death Dis 10:315. doi:10.1038/s41419-019-1540-2 30962426 PMC6453898

[12] Geremia A, Arancibia-Cárcamo CV, Fleming MPP, Rust N, Singh B, Mortensen NJ, Travis SPL, Powrie F. 2011. IL-23–responsive innate lymphoid cells are increased in inflammatory bowel disease. J Exp Med 208:1127–1133. doi:10.1084/jem.20101712 21576383 PMC3173242

[13] Buonocore S, Ahern PP, Uhlig HH, Ivanov II, Littman DR, Maloy KJ, Powrie F. 2010. Innate lymphoid cells drive interleukin-23-dependent innate intestinal pathology. Nature 464:1371–1375. doi:10.1038/nature08949 20393462 PMC3796764

[14] Cox JH, Kljavin NM, Ota N, Leonard J, Roose-Girma M, Diehl L, Ouyang W, Ghilardi N. 2012. Opposing consequences of IL-23 signaling mediated by innate and adaptive cells in chemically induced colitis in mice. Mucosal Immunol 5:99–109. doi:10.1038/mi.2011.54 22089030

[15] Cox J, Ghilardi N. 2011. Dichotomous consequences of IL-23 signaling in chemically induced colitis in mice (59.2). J Immunol 186:59. doi:10.4049/jimmunol.186.Supp.59.2 22089030

[16] Abt MC, Lewis BB, Caballero S, Xiong H, Carter RA, Sušac B, Ling L, Leiner I, Pamer EG. 2015. Innate immune defenses mediated by two ILC subsets are critical for protection against acute Clostridium difficile infection. Cell Host Microbe 18:27–37. doi:10.1016/j.chom.2015.06.011 26159718 PMC4537644

[17] Frisbee AL, Saleh MM, Young MK, Leslie JL, Simpson ME, Abhyankar MM, Cowardin CA, Ma JZ, Pramoonjago P, Turner SD, Liou AP, Buonomo EL, Petri WA. 2019. IL-33 drives group 2 innate lymphoid cell-mediated protection during Clostridium difficile infection. Nat Commun 10:2712. doi:10.1038/s41467-019-10733-9 31221971 PMC6586630

[18] Marafini I, Monteleone I, Di Fusco D, Cupi ML, Paoluzi OA, Colantoni A, Ortenzi A, Izzo R, Vita S, De Luca E, Sica G, Pallone F, Monteleone G, Reeves RK. 2015. TNF-α producing innate lymphoid cells (ILCs) are increased in active celiac disease and contribute to promote intestinal atrophy in mice. PLoS One 10:e0126291. doi:10.1371/journal.pone.0126291 25950701 PMC4423916

[19] Crellin NK, Trifari S, Kaplan CD, Satoh-Takayama N, Di Santo JP, Spits H. 2010. Regulation of cytokine secretion in human CD127+ LTi-like innate lymphoid cells by toll-like receptor 2. Immunity 33:752–764. doi:10.1016/j.immuni.2010.10.012 21055975

[20] Wang R, Cui W, Yang H, Yount J, Collins P. 2023. The interplay between innate lymphoid cells and microbiota. mBio 14:e0039923. doi:10.1128/mbio.00399-23 37318214 PMC10470585

[21] Kim S-H, Cho B-H, Kiyono H, Jang Y-S. 2017. Microbiota-derived butyrate suppresses group 3 innate lymphoid cells in terminal Ileal Peyer’s patches. Sci Rep 7:3980. doi:10.1038/s41598-017-02729-6 28638068 PMC5479798

[22] Fu T, Li Y, Oh TG, Cayabyab F, He N, Tang Q, Coulter S, Truitt M, Medina P, He M, Yu RT, Atkins A, Zheng Y, Liddle C, Downes M, Evans RM. 2022. FXR mediates ILC-intrinsic responses to intestinal inflammation. Proc Natl Acad Sci U S A 119:e2213041119. doi:10.1073/pnas.2213041119 36508655 PMC9907109

[23] Watanabe K, Gilchrist CA, Uddin MJ, Burgess SL, Abhyankar MM, Moonah SN, Noor Z, Donowitz JR, Schneider BN, Arju T, Ahmed E, Kabir M, Alam M, Haque R, Pramoonjago P, Mehrad B, Petri WA. 2017. Microbiome-mediated neutrophil recruitment via CXCR2 and protection from amebic colitis. PLoS Pathog 13:e1006513. doi:10.1371/journal.ppat.1006513 28817707 PMC5560520

[24] Buonomo EL, Cowardin CA, Wilson MG, Saleh MM, Pramoonjago P, Petri WA. 2016. Microbiota-regulated IL-25 increases eosinophil number to provide protection during Clostridium difficile infection. Cell Rep 16:432–443. doi:10.1016/j.celrep.2016.06.007 27346351 PMC4945404

[25] Frisbee AL, Petri WA. 2020. Considering the immune system during fecal microbiota transplantation for Clostridioides difficile infection. Trends Mol Med 26:496–507. doi:10.1016/j.molmed.2020.01.009 32359480 PMC7198612

[26] Shao J, Sheng H. 2010. Amphiregulin promotes intestinal epithelial regeneration: roles of intestinal subepithelial myofibroblasts. Endocrinology 151:3728–3737. doi:10.1210/en.2010-0319 20534719 PMC2940516

[27] Monticelli LA, Osborne LC, Noti M, Tran SV, Zaiss DMW, Artis D. 2015. IL-33 promotes an innate immune pathway of intestinal tissue protection dependent on amphiregulin–EGFR interactions. Proc Natl Acad Sci U S A 112:10762–10767. doi:10.1073/pnas.1509070112 26243875 PMC4553775

[28] Buonomo EL, Madan R, Pramoonjago P, Li L, Okusa MD, Petri WA. 2013. Role of interleukin 23 signaling in Clostridium difficile colitis. J Infect Dis 208:917–920. doi:10.1093/infdis/jit277 23776194 PMC3749013

[29] Wallrapp A, Riesenfeld SJ, Burkett PR, Abdulnour REE, Nyman J, Dionne D, Hofree M, Cuoco MS, Rodman C, Farouq D, Haas BJ, Tickle TL, Trombetta JJ, Baral P, Klose CSN, Mahlakõiv T, Artis D, Rozenblatt-Rosen O, Chiu IM, Levy BD, Kowalczyk MS, Regev A, Kuchroo VK. 2017. The neuropeptide NMU amplifies ILC2-driven allergic lung inflammation. Nature 549:351–356. doi:10.1038/nature24029 28902842 PMC5746044

[30] Gury-BenAri M, Thaiss CA, Serafini N, Winter DR, Giladi A, Lara-Astiaso D, Levy M, Salame TM, Weiner A, David E, Shapiro H, Dori-Bachash M, Pevsner-Fischer M, Lorenzo-Vivas E, Keren-Shaul H, Paul F, Harmelin A, Eberl G, Itzkovitz S, Tanay A, Di Santo JP, Elinav E, Amit I. 2016. The spectrum and regulatory landscape of intestinal innate lymphoid cells are shaped by the microbiome. Cell 166:1231–1246. doi:10.1016/j.cell.2016.07.043 27545347

[31] Zeis P, Lian M, Fan X, Herman JS, Hernandez DC, Gentek R, Elias S, Symowski C, Knöpper K, Peltokangas N, Friedrich C, Doucet-Ladeveze R, Kabat AM, Locksley RM, Voehringer D, Bajenoff M, Rudensky AY, Romagnani C, Grün D, Gasteiger G. 2020. In situ maturation and tissue adaptation of type 2 innate lymphoid cell progenitors. Immunity 53:775–792. doi:10.1016/j.immuni.2020.09.002 33002412 PMC7611573

[32] Peschon JJ, Morrissey PJ, Grabstein KH, Ramsdell FJ, Maraskovsky E, Gliniak BC, Park LS, Ziegler SF, Williams DE, Ware CB, Meyer JD, Davison BL. 1994. Early lymphocyte expansion is severely impaired in interleukin 7 receptor-deficient mice. J Exp Med 180:1955–1960. doi:10.1084/jem.180.5.1955 7964471 PMC2191751

[33] Curio S, Belz GT. 2022. The unique role of innate lymphoid cells in cancer and the hepatic microenvironment. Cell Mol Immunol 19:1012–1029. doi:10.1038/s41423-022-00901-1 35962192 PMC9424527

[34] Lok LSC, Walker JA, Jolin HE, Scanlon ST, Ishii M, Fallon PG, McKenzie ANJ, Clatworthy MR. 2021. Group 2 innate lymphoid cells exhibit tissue-specific dynamic behaviour during type 2 immune responses. Front Immunol 12:711907. doi:10.3389/fimmu.2021.711907 34484215 PMC8415880

[35] Grigg JB, Shanmugavadivu A, Regen T, Parkhurst CN, Ahmed A, Joseph AM, Mazzucco M, Gronke K, Diefenbach A, Eberl G, Vartanian T, Waisman A, Sonnenberg GF. 2021. Antigen-presenting innate lymphoid cells orchestrate neuroinflammation. Nature 600:707–712. doi:10.1038/s41586-021-04136-4 34853467 PMC8702489

[36] Slimings C, Riley TV. 2014. Antibiotics and hospital-acquired Clostridium difficile infection: update of systematic review and meta-analysis. J Antimicrob Chemother 69:881–891. doi:10.1093/jac/dkt477 24324224

[37] Brown KA, Khanafer N, Daneman N, Fisman DN. 2013. Meta-analysis of antibiotics and the risk of community-associated Clostridium difficile infection. Antimicrob Agents Chemother 57:2326–2332. doi:10.1128/AAC.02176-12 23478961 PMC3632900

[38] Littmann ER, Lee J-J, Denny JE, Alam Z, Maslanka JR, Zarin I, Matsuda R, Carter RA, Susac B, Saffern MS, Fett B, Mattei LM, Bittinger K, Abt MC. 2021. Host immunity modulates the efficacy of microbiota transplantation for treatment of Clostridioides difficile infection. Nat Commun 12:755. doi:10.1038/s41467-020-20793-x 33531483 PMC7854624

[39] Nowarski R, Jackson R, Gagliani N, de Zoete MR, Palm NW, Bailis W, Low JS, Harman CCD, Graham M, Elinav E, Flavell RA. 2015. Epithelial IL-18 equilibrium controls barrier function in colitis. Cell 163:1444–1456. doi:10.1016/j.cell.2015.10.072 26638073 PMC4943028

[40] Donlan AN, Simpson ME, Petri WA. 2020. Type 2 cytokines IL-4 and IL-5 reduce severe outcomes from Clostridiodes difficile infection. Anaerobe 66:102275. doi:10.1016/j.anaerobe.2020.102275 32971206 PMC7736165

[41] Wang S, Qu Y, Xia P, Chen Y, Zhu X, Zhang J, Wang G, Tian Y, Ying J, Fan Z. 2020. Transdifferentiation of tumor infiltrating innate lymphoid cells during progression of colorectal cancer. Cell Res 30:610–622. doi:10.1038/s41422-020-0312-y 32367039 PMC7343789

[42] Mackley EC, Houston S, Marriott CL, Halford EE, Lucas B, Cerovic V, Filbey KJ, Maizels RM, Hepworth MR, Sonnenberg GF, Milling S, Withers DR. 2015. CCR7-dependent trafficking of RORγ+ ILCs creates a unique microenvironment within mucosal draining lymph nodes + CCR7-dependent trafficking of RORγ+ ILCs creates a unique microenvironment within mucosal draining lymph nodes. Nat Commun 6:5862. doi:10.1038/ncomms6862 PMC4354100 25575242

[43] Kästele V, Mayer J, Lee ES, Papazian N, Cole JJ, Cerovic V, Belz G, Tomura M, Eberl G, Goodyear C, Maciewicz RA, Wall D, Cupedo T, Withers DR, Milling S. 2021. Intestinal-derived Ilcs migrating in lymph increase IFNγ production in response to Salmonella typhimurium infection. Mucosal Immunol 14:717–727. doi:10.1038/s41385-020-00366-3 33414524 PMC8075955

[44] Huang Y, Mao K, Chen X, Sun M-A, Kawabe T, Li W, Usher N, Zhu J, Urban JF, Paul WE, Germain RN. 2018. S1P-dependent interorgan trafficking of group 2 innate lymphoid cells supports host defense. Science 359:114–119. doi:10.1126/science.aam5809 29302015 PMC6956613

[45] Chen F, Yang W, Huang X, Cao AT, Bilotta AJ, Xiao Y, Sun M, Chen L, Ma C, Liu X, Liu C-G, Yao S, Dann SM, Liu Z, Cong Y. 2018. Neutrophils promote amphiregulin production in intestinal epithelial cells through TGF-β and contribute to intestinal homeostasis. J Immunol 201:2492–2501. doi:10.4049/jimmunol.1800003 30171165 PMC6179911

[46] Minutti CM, Modak RV, Macdonald F, Li F, Smyth DJ, Dorward DA, Blair N, Husovsky C, Muir A, Giampazolias E, Dobie R, Maizels RM, Kendall TJ, Griggs DW, Kopf M, Henderson NC, Zaiss DM. 2019. A macrophage-Pericyte axis directs tissue restoration via amphiregulin-induced transforming growth factor beta activation. Immunity 50:645–654. doi:10.1016/j.immuni.2019.01.008 30770250 PMC6436929

[47] Uddin MJ, Leslie JL, Burgess SL, Oakland N, Thompson B, Abhyankar M, Revilla J, Frisbee A, Donlan AN, Kumar P, Petri Jr WA. 2022. The IL-33-ILC2 pathway protects from amebic colitis. Mucosal Immunology 15:165–175. doi:10.1038/s41385-021-00442-2 34400793 PMC8732277

[48] Ko JH, Kim HJ, Jeong HJ, Lee HJ, Oh JY. 2020. Mesenchymal stem and stromal cells harness macrophage-derived amphiregulin to maintain tissue homeostasis. Cell Rep 30:3806–3820. doi:10.1016/j.celrep.2020.02.062 32187551

