# Supplementary material for: Investigating the impact of antibiotic-induced dysbiosis on protection from Clostridium difficile colitis by mouse colonic innate lymphoid cells
Source: mBio. 2024 Feb 20;15(3):e03338-23. doi: 10.1128/mbio.03338-23 (PMC11209775; doi:10.1128/mbio.03338-23)
Supplement: Supplemental Figures — Figures S1-S6. [file mbio.03338-23-s0001.pdf]

Supplementary Figures

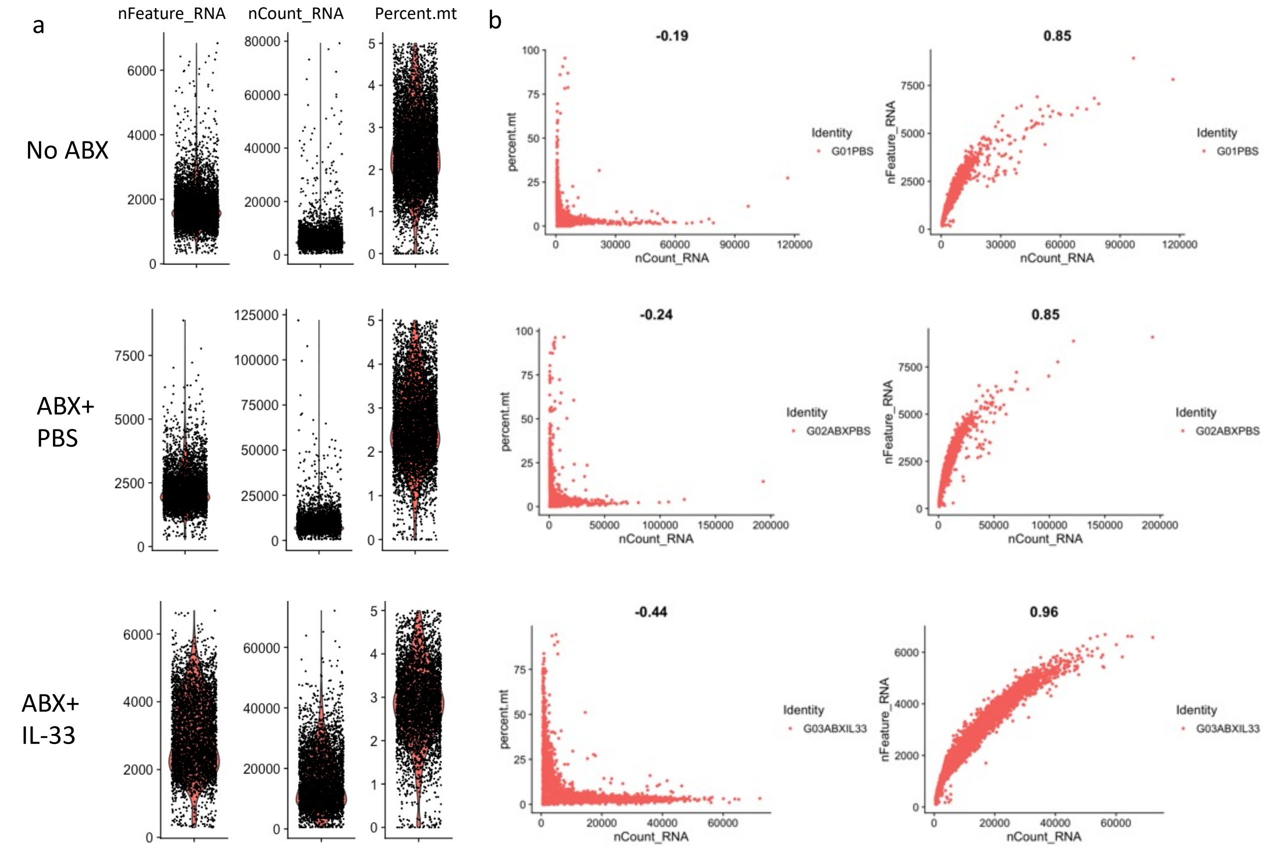

Supplementary Figure 1: Visualization of data QC metrics to filter cells for downstream analysis. a.) The number of unique genes per cell (nFeature_RNA), the total number of molecules detected in each cell (nCount_RNA), and the percent of mitochondrial genes in each cell (Percent,mt) were plotted for each condition. b.) The correlation between the number of transcripts vs. the percent of mitochondrial genes and the number of transcripts vs. the number of unique genes was plotted.

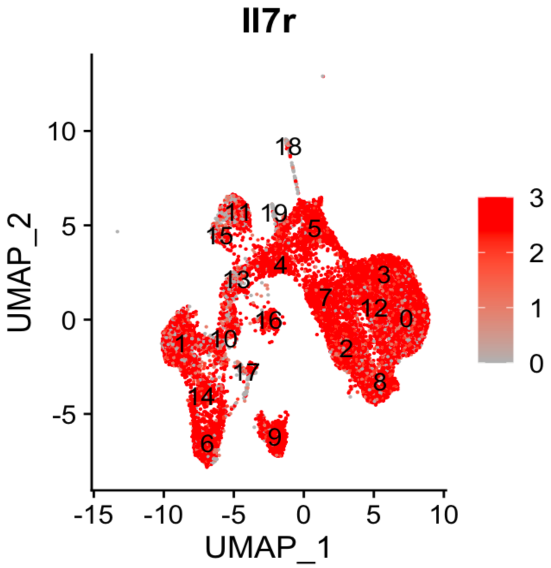

Supplementary Figure 2: Expression of Il7r to determine ILCs and non-ILCs. Expression of Il7r shows all the clusters except clusters 18 and 19 were ILCs.

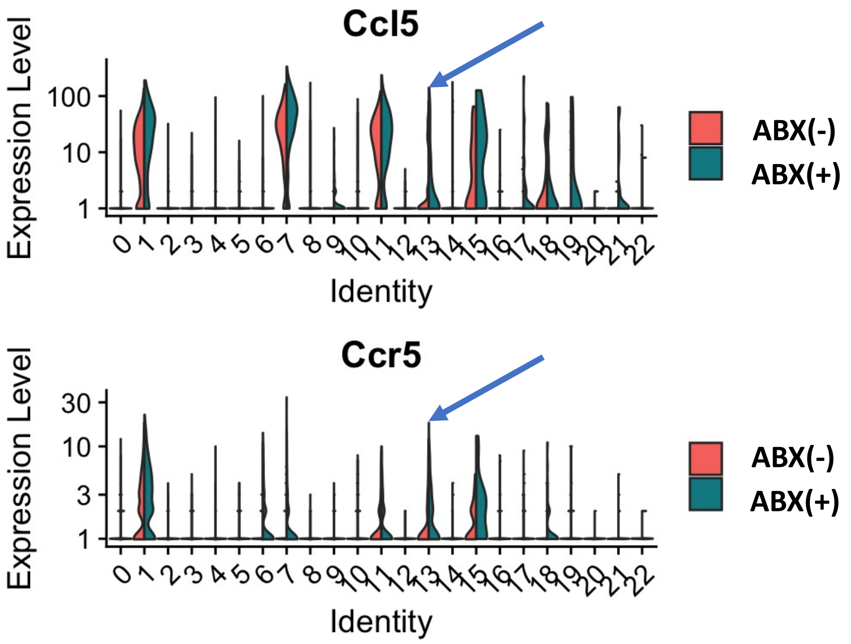

Supplementary Figure 3: Cluster 13 expressed Ccl5 and Ccr5 (related to Figure 2). This figure shows although cluster 13 was classified as an ILC2 cluster, it shared ILC1 genes.

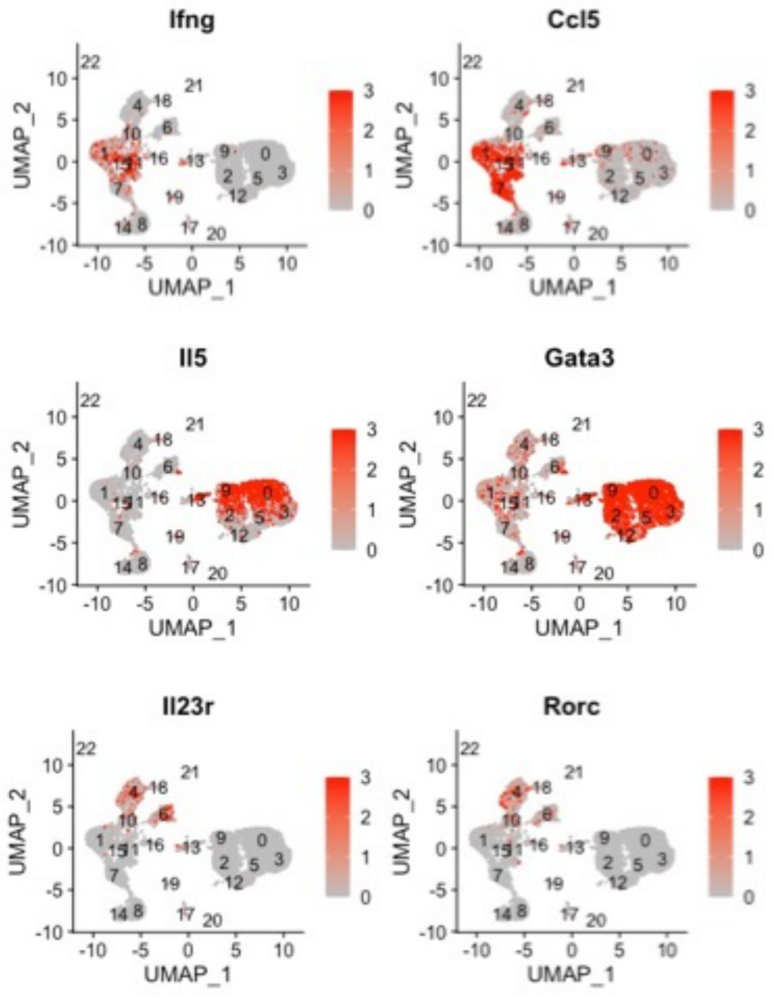

Supplementary Figure 4: Expression profile of key ILC genes. Expression of Ifng, Ccl5, Il5, Gata3, Il23r, and Rorc at ABX (-) and ABX (+) conditions shows ILC clusters. (related to figure 2)

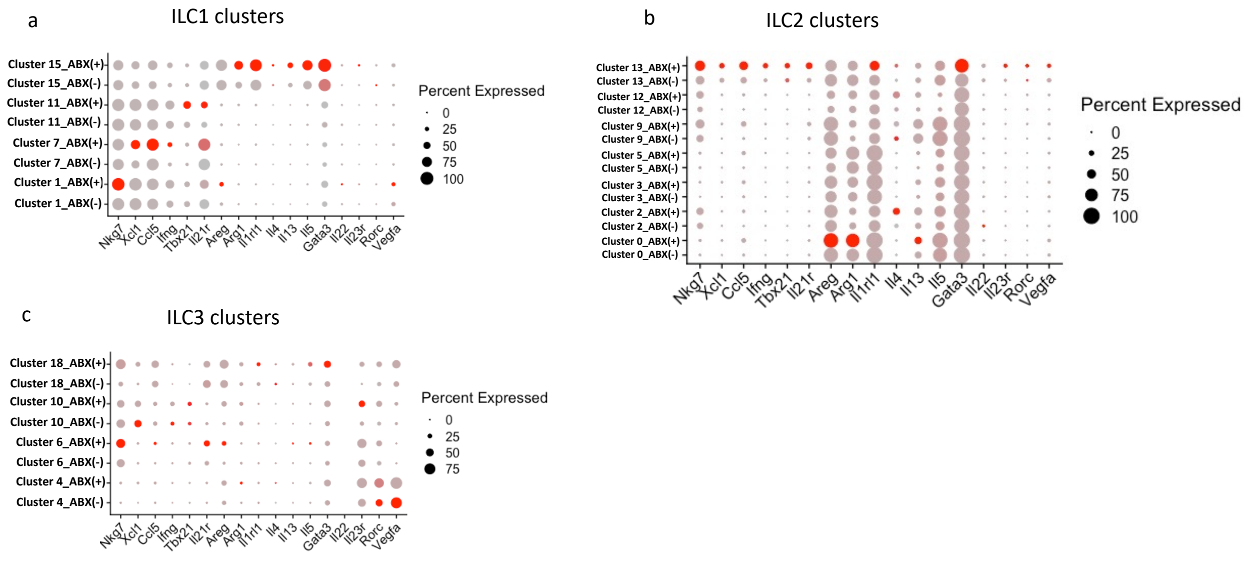

Supplementary Figure 5: Comparison of ILC signature genes in ABX (-) and ABX (+) conditions. X-axis shows gene names; Y-axis shows the cluster identity and treatment conditions. Intensity of the red color is proportional to the average gene expression. ABX, antibiotics.

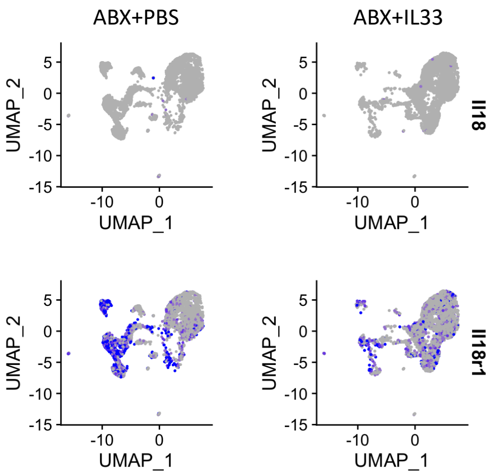

Supplementary Figure 6: ILCs do not express Il18 (top panel). IL-33 treatment downregulated the expression of Il18r1 in colonic ILCs (bottom panel). ABX, antibiotics.
